# Synaptic Size Dynamics as an Effectively Stochastic Process

**DOI:** 10.1371/journal.pcbi.1003846

**Published:** 2014-10-02

**Authors:** Adiel Statman, Maya Kaufman, Amir Minerbi, Noam E. Ziv, Naama Brenner

**Affiliations:** 1 Department of Chemical Engineering, Technion, Haifa, Israel; 2 Network Biology Research Laboratories, Lorry Lokey Center for Life Sciences and Engineering, Technion, Haifa, Israel; 3 Faculty of Medicine, Technion, Haifa, Israel; Hebrew University, Israel

## Abstract

Long-term, repeated measurements of individual synaptic properties have revealed that synapses can undergo significant directed and spontaneous changes over time scales of minutes to weeks. These changes are presumably driven by a large number of activity-dependent and independent molecular processes, yet how these processes integrate to determine the totality of synaptic size remains unknown. Here we propose, as an alternative to detailed, mechanistic descriptions, a statistical approach to synaptic size dynamics. The basic premise of this approach is that the integrated outcome of the myriad of processes that drive synaptic size dynamics are effectively described as a combination of multiplicative and additive processes, both of which are stochastic and taken from distributions parametrically affected by physiological signals. We show that this seemingly simple model, known in probability theory as the Kesten process, can generate rich dynamics which are qualitatively similar to the dynamics of individual glutamatergic synapses recorded in long-term time-lapse experiments in *ex-vivo* cortical networks. Moreover, we show that this stochastic model, which is insensitive to many of its underlying details, quantitatively captures the distributions of synaptic sizes measured in these experiments, the long-term stability of such distributions and their scaling in response to pharmacological manipulations. Finally, we show that the average kinetics of new postsynaptic density formation measured in such experiments is also faithfully captured by the same model. The model thus provides a useful framework for characterizing synapse size dynamics at steady state, during initial formation of such steady states, and during their convergence to new steady states following perturbations. These findings show the strength of a simple low dimensional statistical model to quantitatively describe synapse size dynamics as the integrated result of many underlying complex processes.

## Author Summary

Synapses are specialized sites of cell–cell contact that serve to transmit signals between neurons and their targets, most commonly other neurons. It is widely believed that changes in synaptic properties, driven by prior activity or by other physiological signals, represent a major cellular mechanism by which neuronal networks are modified. Recent experiments show that in addition to directed changes, synaptic sizes also change spontaneously, with dynamics that seem to have strong stochastic components. In spite of these dynamics, however, population distributions of synaptic sizes are remarkably stable, and scale smoothly in response to various perturbations. In this study we show that fundamental aspects of synapse size dynamics are captured remarkably well by a simple statistical model known as the Kesten process: the random-like nature of synaptic size changes; the stability and shape of synaptic size distributions; their scaling following various perturbations; and the kinetics of new synapse formation. These findings indicate that the multiple microscopic processes involved in determining synaptic size combine in such a way that their collective behavior buffers many of the underlying details. The simplicity of the model and its robustness provide a new route for understanding the emergence of invariants at the level of the synaptic population.

## Introduction

Chemical synapses are sites of cell-cell contact specialized for the transmission of signals between neurons and their respective targets. Historically, synapses have been viewed as biological structures that can change when driven to do so by various physiological signals, but are otherwise relatively stable (but see [1]). This view was radically altered, however, by the advent of techniques which allowed for repeated measurements of individual identified synapses in living neurons over long time durations. Such studies have revealed that synapses, in addition to activity-dependent changes in their morphological and functional properties, also change spontaneously in the absence of particular activity patterns, or, for that matter, any activity at all (e.g. [2]–[11]; see also [12]). These spontaneous changes in synaptic properties are not surprising in view of the intense dynamics of synaptic molecules [13]–[18]

Nearly two decades of intensive studies have uncovered a bewildering number of molecules and molecular processes involved in synaptic formation, plasticity and tenacity. While their involvement in aspects of synaptic biology is undeniable, principles of synaptic function often become obscured by the myriad of molecular details (a conundrum raised long ago; see [19]). On the other hand, by accepting the premise that synaptic properties are the integrated result of numerous microscopic processes, which can be heterogeneous, non-stationary, stochastic, and to some extent intractable, repeated measurements of the properties of individual synapses provide an opportunity for quantitative, phenomenological study of long-term *population dynamics* of synapses. This is essentially a statistical approach in which the dynamics of the individual synapse are described probabilistically, while causal or deterministic relations emerge at the population level. Such studies can uncover overarching principles that govern synaptic population properties as well as their relationships with physiological signals such as network activity [3]–[5] and neuromodulation [20]. Indeed, recent work based on such measurements has resulted in several key findings, described in more detail below: (1) distributions of synaptic sizes are broad, skewed and remarkably stable over time; (2) individual synapse sizes exhibit significant spontaneous fluctuations over time scales of many hours; and (3) these synaptic dynamics are size-dependent and constrained by network activity and other physiological signals.

In the current study we use a simple and well known statistical model, the Kesten process, to describe effectively synaptic remodeling dynamics based on the three aforementioned findings. We use empirical data from continuous, long-term (days) imaging experiments to show that the model captures the dynamics of individual synapses and the statistical properties of synaptic populations, the effects of network activity levels and cholinergic tone and the dynamics of synapse formation.

## Results

### Rationale and underlying experimental basis of the proposed model

**Figure 1:**
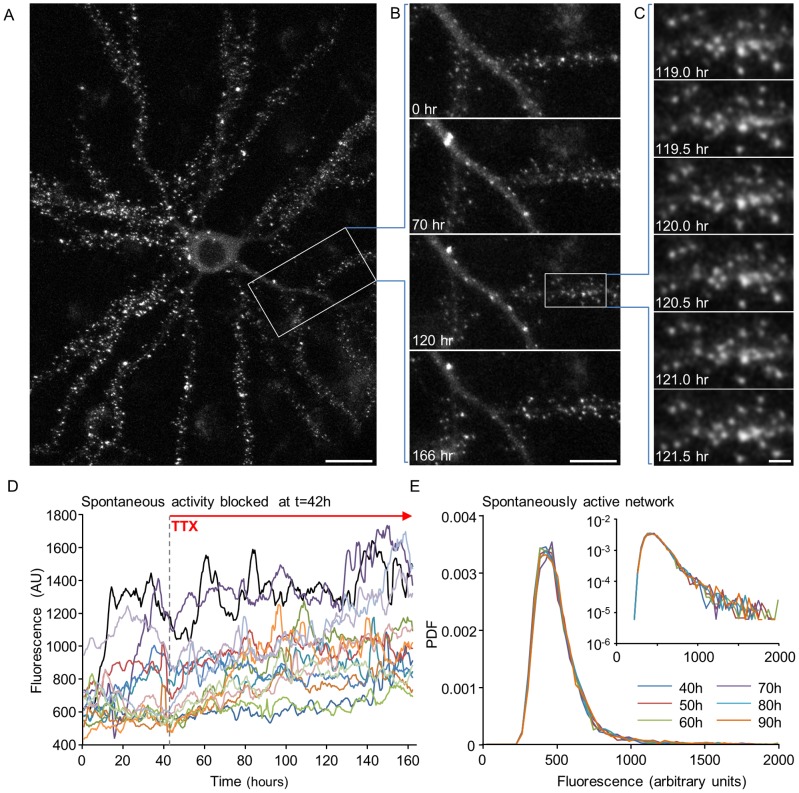
Synaptic remodeling and synaptic size distributions in long-term experiments. (**A**) A single neuron expressing PSD-95:EGFP. Fluorescent puncta represent postsynaptic sites formed on dendritic spines and shafts. (**B**) A 7-day time-lapse series (30 min intervals, or 48 images/day) of the region enclosed in a rectangle in **A**. Only a small subset of the data is shown here. (**C**) Magnification of region enclosed in a rectangle in **B**, demonstrating the actual temporal and spatial resolution of imaging data collected in these experiments. All images in panels **A–C** are maximal intensity projections of 9 images collected at 9 focal planes spaced 0.8 µm apart. Bars: **A**, 20 µm; **B**, 10 µm; **C**, 2 µm. (**D**) Fluorescence of synapses as a function of time (“synaptic trajectories”; measurement taken every 30 min) of 16 synapses in a spontaneously active network to which TTX was added at t = 42 hours. Data is shown after smoothing with a 5 time point window to decrease the effects of measurement noise, and after normalizing the fluorescence values by the average over the entire experiment. (**E**) Probability density function (PDF) of PSD-95:EGFP puncta fluorescence values at 10 hour intervals in a spontaneously active network. Inset: the same data on semi-logarithmic axes.

We base our model on empirical findings that were obtained in a previously described system [4], [20] in which *ex-vivo* networks of rat cortical neurons, automated microscopy, multielectrode array (MEA) recordings of network activity, fluorescent reporters and provisions for maintaining optimized environmental conditions were combined to allow for imaging and tracking of individual synapses at 10–30 min intervals for many days (as shown in Figs. 1A–C). Sizes of individual glutamatergic synapses were estimated by quantifying the fluorescence of enhanced green fluorescent protein tagged PSD-95 (PSD-95:EGFP). PSD-95 is a core postsynaptic scaffolding protein in glutamatergic synapses that is thought to control the number of glutamate receptors at the postsynaptic membrane through direct and indirect interactions [21], [22]. Therefore PSD-95:EGFP fluorescence can serve as a proxy of synaptic strength [23]. More conservatively, changes in PSD-95:EGFP fluorescence reflect synaptic remodeling, changes in spine head size and PSD area [24], [25] (and will be referred to hereafter as synaptic size). Previous work with this system as well as studies from other groups using different systems, gave rise to three key findings that form the basis of our model. We now summarize these in some detail:

#### (1) Distributions of synaptic sizes are broad and stable over time

Imaging based estimations of synaptic sizes (e.g. [3]–[5], [20]) and electrophysiological measurements of synaptic connection strengths (e.g. [26], [27]) have shown that distributions of synaptic strengths are broad and highly skewed. It has been suggested that these are consistent with a log-normal distributions [3], [27], [28], a matter that will be revisited later. This distribution is illustrated in Fig. 1E for PSD-95:EGFP based estimates of synaptic sizes. Importantly, in spontaneously active networks, this distribution remains remarkably stable for many days as long as network activity is not perturbed (Fig. 1E; [4], [20]).

#### (2) Individual synapses exhibit spontaneous remodeling

When individual synapses are tracked for several days, it becomes apparent that their sizes fluctuate significantly (e.g. [2]–[8], [24]). Moreover, a considerable component of this spontaneous remodeling appears to be activity independent [2], [4], [5]. This is illustrated in Fig. 1D, showing the fluorescence of individual synapses over time (“synaptic trajectories”) before and after application of Tetrodotoxin (TTX), a potent blocker of voltage dependent sodium channels, which suppresses practically all spontaneous network activity. All data shown in this figure was first “smoothed” with a 5 time point low pass filter, and thus the fluctuations in synaptic size seen here and elsewhere cannot be solely attributed to measurement noise (for a detailed analysis see Fig. S1). Interestingly, spontaneous changes in connection strengths occurring over comparable time scales have also been observed by electrophysiological recordings (e.g. [11]).

#### (3) Synaptic change is a decreasing function of synaptic size

**Figure 2:**
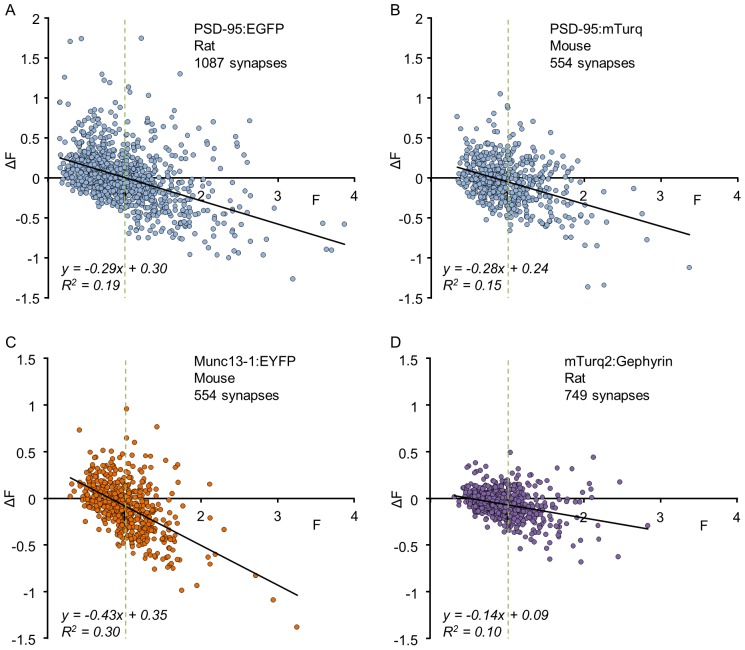
Changes in the fluorescence of individual synapses as a function of their initial fluorescence. Each dot represents one synapse. *ΔF* represents the change in fluorescence after a given time interval. Data were normalized by dividing the fluorescence of each synapse by the average fluorescence of all synapses at time t = 0 to allow pooling of data from multiple neurons irrespective of some variability in neuron-to-neuron expression levels. Solid lines are linear fits; vertical dashed lines highlight the average synaptic size ( = 1, after normalization). All data was obtained under baseline conditions from unperturbed networks. (**A**) Rat cortical neurons expressing PSD-95:EGFP; 1087 synapses from 10 neurons in 5 separate experiments. Images were collected at 30 min intervals; ΔF was measured after a 24 hour interval (see ref [20] for further details). (**B**) Mouse cortical neurons expressing PSD-95:mTurquoise; 554 synapses from 8 neurons in 6 separate experiments. Images were collected at 25 min intervals; *ΔF* was measured after a 15 hour interval (see ref [63] for further details). (**C**) Mouse cortical neurons expressing munc13-1:EYFP; 554 synapses from 8 neurons in 6 separate experiments. Imaging was performed as in B (see ref [63] for further details). (**D**) Rat cortical neurons expressing mTurquoise2:Gephyrin; 749 synapses from 27 neurons in 4 experiments. Images were collected at 60 min intervals; ΔF was measured after a 24 hour interval (Anna Rubinski and Noam E. Ziv, unpublished data).

The stable distributions of synaptic sizes and the exuberant spontaneous remodeling dynamics of individual synapses would seem to be at odds, unless spontaneous remodeling is somehow confined. Indeed, when changes in synaptic sizes are plotted as a function of initial synaptic size it is seen that large synapses tend to grow smaller on average, whereas small synapses tend to grow larger [4], [5], [20]. As shown in Fig. 2, this dependence was observed when fluorescently tagged molecules (PSD-95, Munc13-1, Gephyrin), targeted specifically to pre- or postsynaptic compartments or to excitatory or inhibitory synapses, were followed over 15–24 hours in rat or mouse cortical neurons. The size dependence is qualitatively robust: in all these experiments plotting the change in synaptic size as a function of initial size reveals a negative correlation between the two, which can be fitted to a line that crosses the abscissa at the average synaptic size (defined to be 1 in these data by normalization). Here too, similar tendencies were also observed in electrophysiological measurements of synaptic strengths repeated at 12 hour intervals [11].

The implication of these data is that synaptic sizes are not just fluctuating as a simple random walk; changes in synaptic size depend on current synaptic size. As a result, synaptic sizes are constrained and distributed around some mean value. In the next section we use this observation to construct a minimal stochastic model to describe such a process.

### Synaptic dynamics as a Kesten process

Synaptic size is affected by multiple molecular mechanisms of a variety of natures: direct and indirect, size-dependent and -independent, activity dependent and -independent, which collectively span a broad range of timescales [18], [29]. The integrated effect of these various mechanisms support the long-term stability of synaptic structure but also result in rich dynamics over multiple timescales. From a practical point of view, changes in synaptic size can be broadly divided into two types: additive, namely independent of current synaptic size, and size-dependent, of which the simplest dependence is linear – namely multiplicative changes. The question of whether various forms of synaptic plasticity are additive or multiplicative has received considerable attention in the literature [28], [30]–[32]. From general biological considerations, however, it is plausible to expect that over long enough times synapses will undergo both types of changes. Indeed, on the basis of plots such as that shown in Fig. 2 it has been suggested that individual synaptic dynamics are the sum of three components [20]: two deterministic components - a multiplicative downscaling and an additive positive term - with an added stochastic component (see Fig. 5G in [20]). Here, we propose a model of synaptic dynamics which is inherently stochastic, includes both additive and multiplicative random components, and relates to electrical activity through a parametric dependence of the stochastic processes.

We model the synaptic size trajectories by the following dynamics:
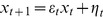
(1)Where *x_t_* is the synaptic size at time *t* and *ε_t_* and *η_t_* are random variables drawn from some distribution. This is a minimalistic model that includes both additive and multiplicative random events; it is an effective description in which each variable does not necessarily relate directly to a microscopic event but rather captures the integrated effect of many processes as discussed in the Introduction (see Supporting Information, Text S1, for a more detailed justification). It is formulated here in discrete time, so that the random variables represent all processes that occurred in the time between two measurements; therefore, if measurements are made at a different time resolution, the effective variables *ε_t_* and *η_t_* will generally be altered. Accordingly, we focus here on general properties of the model and not on fitting precise, absolute values to the variables.

In the simplest form of the model the variables *ε_t_*, *η_t_* are drawn independently at each time step and independently from one another, each from a given (fixed) distribution. Note that *ε_t_* is drawn from a distribution that generally includes values smaller or larger than 1, so that this factor can either decrease or increase synaptic strength.

In probability theory the model (1) is known as the *Kesten process* [33] and has been used to describe complex systems in economics and other fields [34], [35]. In spite of its seemingly simple formulation, it can give rise to rich and complex dynamics.

**Figure 3:**
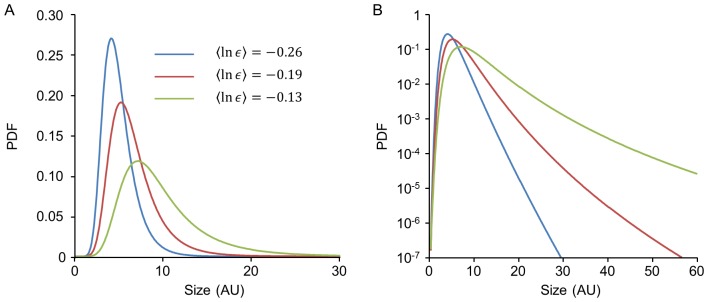
Limiting distributions of the Kesten process in the stable regime. (**A**) Simulations for three different distributions of *ε* corresponding to different values of 〈ln *ε*〉 with the distribution of *η* held fixed. Note that as 〈ln *ε*〉 approaches 0 the limiting distribution approaches the instability boundary and broadens. (**B**) Same distributions as in A plotted on a semi-logarithmic scale.

The Kesten process is known to exhibit two qualitatively different behaviors depending on the regime of the crucial parameter 〈ln *ε*〉, the average logarithm of ε over its distribution: For 〈ln *ε*〉>0 the process diverges and no limiting distribution is reached. For 〈ln *ε*〉<0 it is statistically stable and approaches a limiting distribution *f(x)* at long times. Some intuition for this property can be gained by considering the case *η_t_ = 0* in (1): the process then reduces to a purely multiplicative one. In this case, ln *x* performs a random walk with steps of size ln *ε*. If the mean step-size is positive, 〈ln *ε*〉>0, the mean of the random walk drifts to infinity; adding *η_t_* cannot prevent this runaway. If, on the other hand, the mean step-size is negative, 〈ln *ε*〉<0, the logarithmic random walk tends to -∞ and accordingly the original variable *x_t_* decreases to zero. In this case the “injection” of a positive (on average) *η_t_* in each step can provide the balancing drive away from zero required to induce a finite average and a stable limiting distribution [35]. This is indeed the case, and within this region, a stable limiting distribution exists regardless of the distribution of *ε* and *η*. This limiting distribution is generally non-Gaussian, skewed and decays asymptotically as a power-law [33]. It follows that in the stable regime 〈ln *ε*〉<0 the Kesten process exhibits the qualitative features of our data: fluctuating individual trajectories accompanied by skewed, non-Gaussian stable distributions. As 〈ln *ε*〉 increases and the process approaches the instability transition from below, although it remains stable, it changes quantitatively: trajectories exhibit larger and larger excursions to rare values (“intermittent”-like behavior), and correspondingly the stable limiting distribution broadens (as illustrated in Fig. 3).

We first characterized the regime of parameters of the model which is in quantitative agreement with our data. Naively, one might expect that a simple linear regression of the empirical mapping *x_t_*_+1_ = *ε_t_x_t_*+*η_t_* would give an estimate of the first moments of the *ε*- and *η-* distributions. Such estimations, however, prove to be highly noisy and unreliable, as the small changes in synapse sizes (or more specifically, PSD-95:EGFP fluorescence levels) measured over these short time intervals are dominated by measurement noise, as shown by measuring ‘changes’ in chemically fixed synapses (Fig. S1; 1067 synapses from 4 neurons). Much of this measurement noise is removed by filtering the data with a 5 time-point low pass filter (Fig. S1B–D), but this procedure precludes estimates based on single time steps. We therefore estimated the average values of *ε* and *η* from iterated mappings over multiple time steps (i.e. longer time intervals) and multiple synapses under the assumption that these values are stationary and similar for all synapses. This estimation is based on the following observation:

When the Kesten mapping is applied twice consecutively one finds the relation
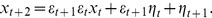
(2)


Repeated application of this relation and averaging over the distributions of the random variables given *x_t_* results in an explicit formula for the average k-iterated map
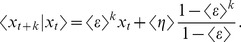
(3)


**Figure 4:**
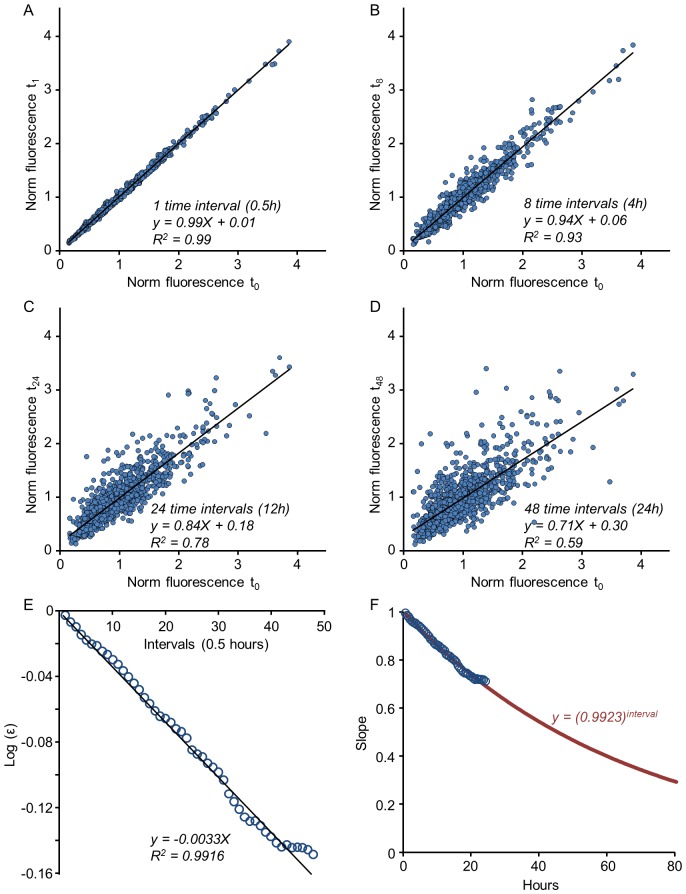
Estimating Kesten parameters in experimental data. An estimate of the parameter 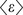 can be obtained from k-times iterated mappings of the data as explained in text. These mappings are shown for 1, 8, 24 and 48 time-steps, corresponding to 0.5, 4, 12 and 24 hours respectively (**A–D**); from each such mapping the slope of the linear regression (solid black line) is extracted. (**E**) The logarithmic values of these slopes (circles) plotted as a function of iteration number and fit by linear regression (solid black line) to obtain an estimate of 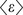. (**F**) The measured slopes (circles) with the predicted slope values (red line) over an extended time scale.

Estimates of 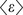 could therefore be obtained by applying linear regressions to such mappings over an increasing number of time steps k, and fitting the slopes to a power k of 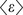. This procedure is illustrated in Fig. 4: Fig. 4A depicts a linear fit for a one-step empirical mapping (k = 1; 1087 synapses tagged with PSD-95:EGFP; same low pass filtered data as in Fig. 2A), whereas Figs. 4B–D show mappings for 8, 24 and 48-steps, respectively. As expected, these mappings become more noisy as the number of steps increases; at the same time, the slopes of the linear fits decrease, corresponding to an increasing value of k and the reduced value of 〈*ε*〉*^k^* (reminder: in the stable regime approximately 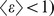. As shown in Figs. 4E,F for 1087 synapses followed for 24 hours (48 time steps of 30 min) this procedure allowed for a reasonable estimation of 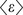, which in this case was found to be 0.9923. We validated this procedure by using only half of the data to estimate 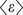 and then using this estimate (0.9925) to predict linear fit slope values for increasing k values in the second half of the data. As shown in Fig. S2A, the prediction was quite good.

These estimates are very close to 1, indicating that the process may be near the transition point (formally 〈ln ε〉 = 0), consistent with the distributions being broad and long-tailed. We used the same procedure on the data of Fig. 2B–D and found similar estimates (0.9899, 0.9829, 0.9934 for time steps of 25, 25 and 60 min, respectively).

The second parameter in Eq. (3), 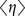, sets the scale of the population average. In the analysis presented in Fig. 4 the data was normalized to unit mean at t = 0; As the mean synapse size remains constant (Fig. 1E) the value of 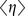 in these units is constrained to be 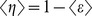. Consequently, equation 3 is reduced to
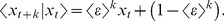
(4)Indeed the values of the constant term in the aforementioned linear fits come out very close to 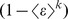 not only for the four time points shown in Fig. 4A–D but also for the linear fits performed at all 48 time points (Video S1).

**Figure 5:**
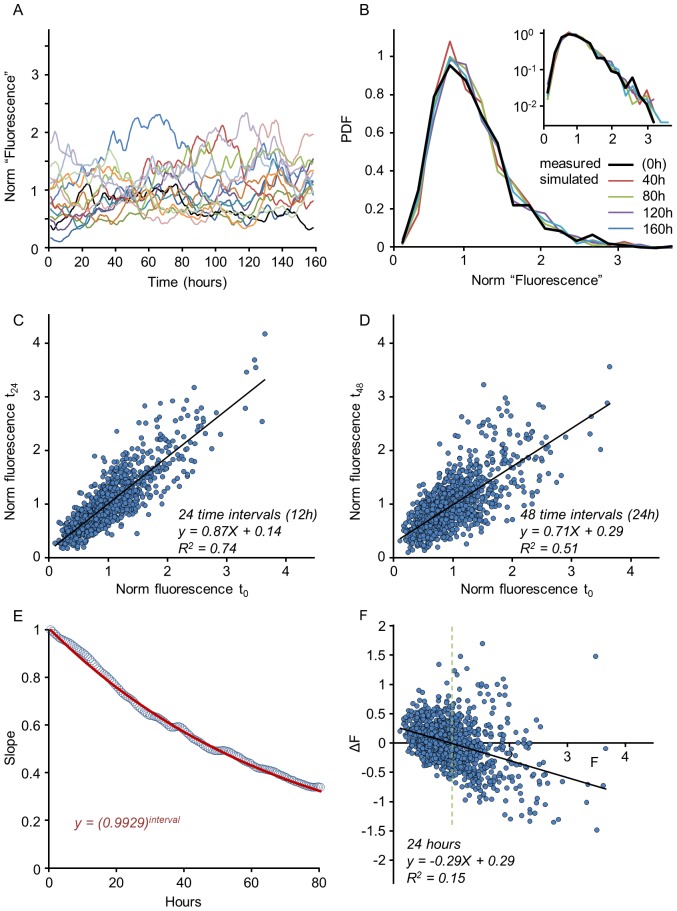
Properties of the Kesten process in estimated parameter regime. **(A)** Simulated synaptic trajectories of 14 out of 1075 synapses, evolved for 160 hours at 30 min intervals. Synapses were sorted according to initial size and then every 76^th^ trajectory was selected for display (compare with Fig. 1D). The Kesten process parameters used here were based on the estimate shown in Fig. 4 (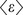 = 0.9923±0.05; 〈*η*〉 = 0.0077±0.03) and values were obtained from Gaussian distributions with these parameters. The initial data set (1087 synapses) was identical to that shown in Figs. 2A and 4; 12 synapses were ‘lost’ during the simulation (i.e. their values reduced to 0) and were excluded from subsequent analysis. (**B**) Synaptic distributions along time, starting from a measured distribution (thick black line) and applying the time evolution of the Kesten process to this initial population. Four subsequent time points are plotted as indicated. Inset shows the same distributions on a semi-logarithmic scale. (**C,D**) Examples of k-times iterated mappings corresponding to 24 and 48 time-steps (compare with Fig. 4C,D). (**E**) Slope of k-times iterated mappings as a function of k in simulated trajectories (circles) and in a theoretical prediction based on Eq. (3) (red solid line, red equation). (**F**) Scatter plot of changes in synapse size as a function of initial size for simulated trajectories for the period covering first 24 hours of the simulation. Note the strong resemblance with the experimental measurements of Fig. 2A.

We next tested the ability of the Kesten process in the estimated parameter regime to faithfully reproduce the experimentally measured dynamics of individual synapses, the distribution of synaptic sizes and the relationships between changes in synaptic size and initial size. The results are shown in Fig. 5. Using the initial distribution of synapses from Fig. 2A and the estimate of 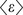 derived in Fig. 4, trajectories were simulated for all 1087 synapses for 320 half-hour time steps (160 hours). As shown in Fig. 5A, the ‘sizes’ of individual simulated synapses fluctuated in a manner qualitatively similar to that observed for real synapses (compare with Fig. 1D). Interestingly, in common with experimental observations the simulation was associated with the ‘elimination’ of a small number of synapses, i.e. synapses whose ‘size’ dropped to zero. Such synapses (12 out of 1087 in this example) were not included in subsequent analysis. The distribution of simulated synaptic sizes remained stable and similar to the original, experimentally measured, skewed distribution for the entire simulation period (Fig. 5B). When the slopes of linear regressions were used to estimate 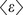 as explained for Fig. 4, the resulting estimate (0.9929; Fig. 5C–E) was very close to the one used for the simulation (0.9923; Fig. 4) validating this approach to estimate 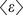. Finally, when changes in synaptic ‘sizes’ after the first 24 hours were plotted as a function of their original ‘sizes’ the resulting dependence was remarkably similar to that observed experimentally (compare Fig. 5F with Fig. 2A).

While the methods described above can give good estimates of the first moments (means) of *ε* and *η*, they do not provide information on their second moments (variances). In the simulation described above, values for the latter were chosen such that the decay rate of goodness of fit (R^2^ values in plots such as those of Fig. 5C,D) was similar to that observed for the experimental data. In principal, the standard deviations of *ε* and *η* could be directly estimated from the squares of residuals in a linear regression of the mapping *x_t_*_+1_ = *ε_t_x_t_*+*η_t_* (see legend of Fig. S2). However, as noted above, apparent changes in synapse sizes measured over single time steps were dominated by measurement noise, effectively ruling out direct estimations of standard deviations in this manner. Nevertheless, when such estimations were obtained and compared for low-pass filtered experimental and simulated data sets, they were quite similar (Fig. S2B,C) indicating that the standard deviation values used in the simulation above were reasonable.

These results thus show the Kesten process can quantitatively capture and faithfully reproduce the dynamics of individual synapses and the distributions of synaptic sizes in large populations of dynamic synapses.

### Sensitivity of the Kesten process to parameters

**Figure 6:**
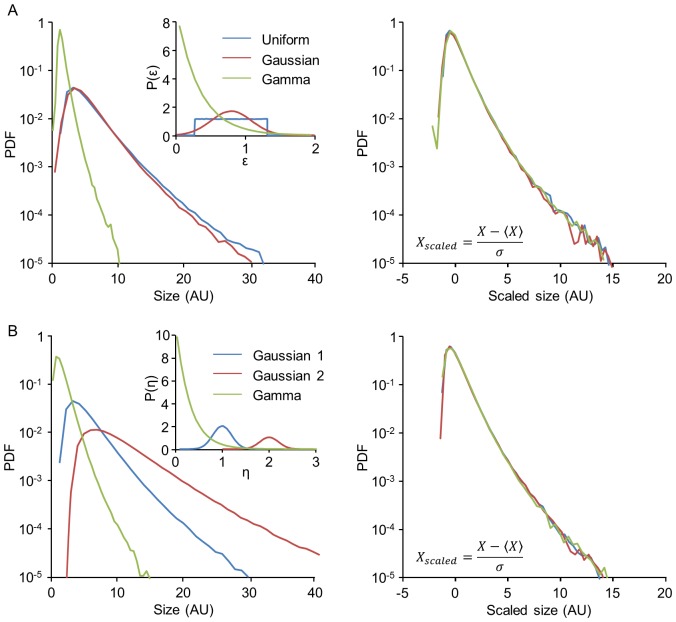
Invariance of Kesten limiting distribution shape to different ε- and η- distributions. (**A**) Simulated limiting distributions of Kesten processes with the three different ε-distributions shown in inset, all belonging to the same μ-class 6, that is, 〈*ε* ^6^〉 = 1. The distribution of η was held fixed. The same three distributions after scaling are shown on the right. (**B**) Simulated limiting distributions of Kesten processes with the three different η-distributions shown in the inset. The distribution of *ε* was held fixed. The same three distributions after scaling are shown on the right.

Being a phenomenological model, the question naturally arises how sensitive is the fit of experimental data to the parameters of the model. The answer to this question is largely determined by the sensitivity of the Kesten process itself to the underlying distributions from which *ε*, *η* are drawn. In his original work, Kesten showed that the tail of the limiting distribution, when it exists in the stable region, always decreases asymptotically as a power-law [33]:
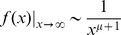
(5)where *μ* is a property of the *ε*-distribution. It is the positive number that obeys the relation 〈*ε^μ^*〉 = 1; thus *μ* depends on all moments of the distribution and is not unique, as many different distributions can have the same value of *μ* satisfying this relation. This suggests that different *ε*-distributions belonging to the same *μ*-class, when used in Kesten processes, may give rise to similar limiting distributions. Indeed, simulations of the Kesten process displayed in Fig. 6 support the possibility that these limiting distributions are in fact of identical shape. Fig. 6A shows that three members of such a *μ*-class, with very different *ε*-distribution types (Uniform, Gaussian and Gamma distributions), result in limiting distributions of the Kesten process with identical shapes when scaled linearly (right panel), not only in their asymptotic tail but over their entire range. This implies that the distribution *shape* is robust with respect to the details of *ε* within a given *μ*-class.

Not only is the Kesten process quite insensitive to the particular choice of the *ε* -distribution, we found that its limiting distribution is insensitive also to the additive random variable *η*, except in determining the absolute scale. In other words, limiting distributions of Kesten processes can be effectively scaled merely by changing the distribution of the random variable *η*. As explained above, intuitively the role of this variable is to provide an effective boundary condition for the multiplicative process, keeping synaptic sizes from collapsing to zero; accordingly, it does not affect the distribution shape but only the absolute set-point at which the “forces” balance each other. This property is illustrated in Fig. 6B, where the *ε*-distribution was held fixed and different distributions were assigned to *η*. This figure shows the resulting limiting Kesten distributions (left), as well as the same distributions on a normalized scale (right), showing that they all have the same shape.

The conclusion from these results is that the same population distribution can be obtained from the Kesten process with many underlying sets of microscopic random variables (*ε* and *η*). On one hand, this insensitivity to microscopic details strongly justifies its usefulness as an effective description of the phenomenon studied here; on the other, this robustness implies that by measuring the distribution alone one cannot infer much about the details of these underlying processes [36].

### Scaling of synaptic distributions through changing conditions

The analysis described so far indicates that synaptic size dynamics governed by a stochastic Kesten process result in synaptic populations with limiting size distributions which are qualitatively and quantitatively similar to empirically measured distributions of synaptic sizes. Can this statistical framework also explain *changes* in synaptic size distributions caused by various experimental manipulations?

**Figure 7:**
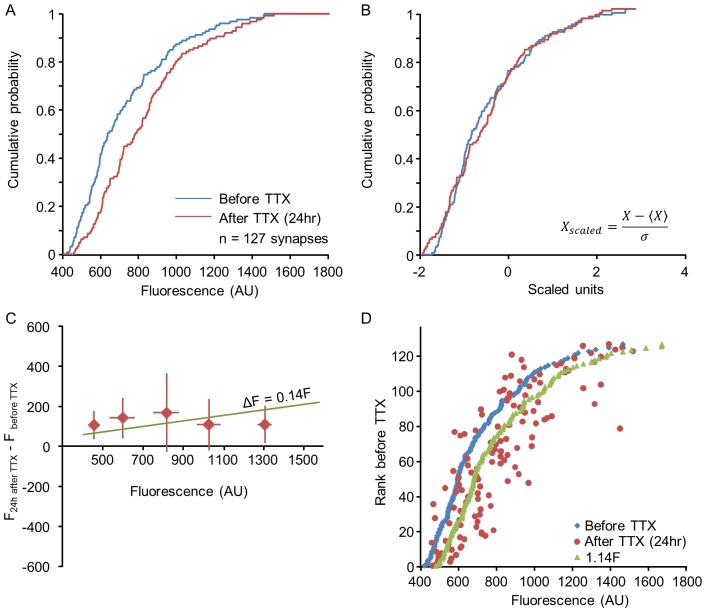
Scaling of synaptic size distributions following application of TTX. (**A**) Cumulative distribution of synaptic sizes all belonging to a single neuron, before (blue), and 24 hours after (red) applying TTX, which suppresses all spontaneous network activity. (**B**) Same distributions shown in A after scaling. (**C**) Changes in the fluorescence of individual synapses (*ΔF*) during the first 24 hours after TTX addition (averages and standard deviations of binned data) reveal no particular relationships with their initial size (*F*). The green line represents the expected relationships between *ΔF* and F had sizes of individual synapses scaled through multiplication by 1.14 (the ratio of mean synaptic size before and after TTX addition). (**D**) Scaling of synaptic size distributions does not preserve rank order. Synapses were sorted according to their size before TTX addition and plotted according to their original sizes (blue dots). The sizes of the same synapses 24 hours after TTX addition are shown as red dots. Note the significant departure from the original rank order. Expected synaptic rank order vs. new size, had synapse growth followed individual multiplicative scaling, is shown as green dots. Data from one of the two neurons shown in Figs. 1E–G.

**Figure 8:**
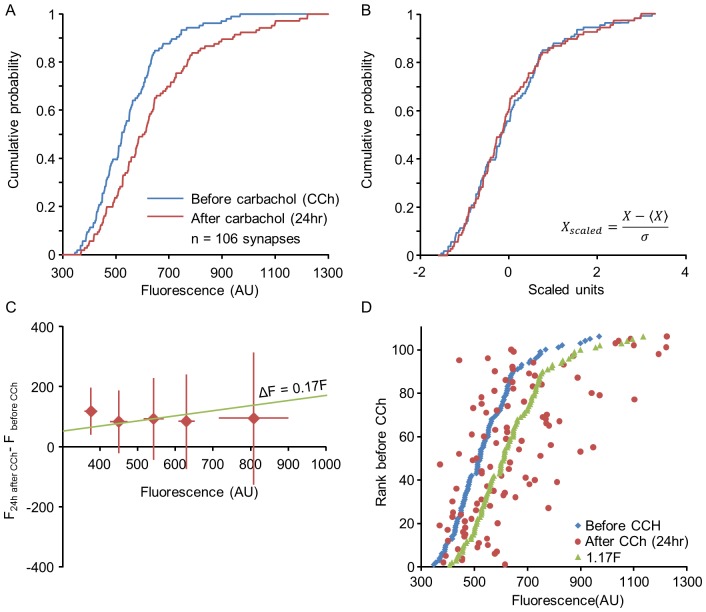
Scaling of synaptic size distributions following application of CCh. (**A**) Cumulative distribution of synaptic sizes all belonging to a single neuron, before (blue), and 24 hours after (red) exposure to CCh. (**B**) Same distributions shown in A after scaling. (**C**) Changes in the fluorescence of individual synapses (*ΔF*) during the first 24 hours after CCh addition (averages and standard deviations of binned data) reveal no particular relationships with their initial size (*F*). The green line represents the expected relationships between *ΔF* and *F* had sizes of individual synapses scaled through multiplication by 1.17 (the ratio of mean synaptic size before and after CCh addition). (**D**) Scaling does not preserve rank order. Synapses were sorted according to their size before CCh addition and plotted according to their original sizes (blue dots). The sizes of the same synapses 24 hours after exposure to CCh are shown as red dots. Note the significant departure from the original rank order. Expected synaptic sizes, had synapse growth followed simple multiplicative scaling, are shown as green dots. All data obtained from one neuron.

Previous studies have shown that statistical properties of synaptic populations are affected by changes in network activity as well as by additional experimental perturbations. In the most well-known example, pharmacological suppression of network activity by TTX leads to a broadening of synaptic size distributions [4], [37]; a similar effect was observed following experimental elevation of cholinergic tone using carbachol (CCh), which did not strongly alter mean firing rates but changed the temporal structure of spontaneous activity [20]. Following TTX application, synaptic distributions were previously shown to retain their shape in scaled units, and so this phenomenon was referred to as “synaptic scaling” (reviewed in [38]). This property is found also in our measurements of synaptic sizes: Fig. 7A shows the distribution of synaptic sizes measured for one neuron, before and 24 hours after the application of TTX. Suppression of spontaneous activity was associated with a broadening of synaptic size distribution. Fig. 7B shows the same distributions on a scaled axis: 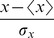 (this variable, which measures the number of standard deviations away from the mean, is sometimes referred to as the z-score). It is seen that the distribution shape remained intact. The same scaling behavior is seen also following exposure to CCh (Fig. 8A,B). Thus the scaling of synaptic size distributions – a change in the distribution scale but not in its shape – is found also in response to a more general perturbation that does not significantly change average firing rates.

Scaling, or “data collapse”, of distributions is a well-known phenomenon in the physics of complex systems [39]–[41], and has recently been observed also in biological fluctuations [36]. In order to understand the origin of scaling in synaptic size distributions following a perturbation, it is helpful to observe individual synapse dynamics before, during and after the perturbation, as our time-lapse measurements allow for. We have seen in the previous sections that individual synapses exhibit what appear to be stochastic trajectories over time, and our aim is now to reconcile these dynamics with the rescaling property at the population level.

First, we consider the relation between the initial sizes of individual synapses and their size change 24 hours after performing a perturbation *Δx* = *x_t = 24 h_−x_t = 0 h_*. Figs. 7C and 8C depict this change averaged over synapses as a function of the initial value *x_t = 0 h_*, showing that there is no correlation between initial synaptic size and the change in its size induced by the perturbation. Second, one may use the rank order of individual synapses before and after the perturbation to investigate the transformation they have undergone: any deterministic, monotonically increasing transformation acting on individual synapses would preserve their rank order in the population. Figs. 7D and 8D show the rank orders prior to the perturbations as a function of value (blue dots), tracing a curve with the same shape as that of the cumulative probability distribution. In the same figure, the final values after perturbation are depicted as a function of their original rank order (red dots); this analysis clearly shows that rank order is not preserved even though the distribution exhibits scaling. This result corroborates previous work which quantified the change in rank order over time within steady experimental conditions, and showed that the rank order gradually deteriorates even under conditions where the distribution remains exactly the same [4].

In principle, one could induce a scaling of the distribution by simply multiplying all synapses by a constant such that for each synapse 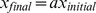; this was the interpretation originally given to the population-level data [37]. At the individual synapse level, this would imply a synaptic change (*Δx*) which increases linearly with the initial value such that 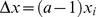 (with *a>1* for a broadening of the distribution, as in these experiments), and the preservation of rank order. Both these predictions are inconsistent with our single synapse measurements (the result of such a transformation on the original data is illustrated in Figs. 7C,D and 8C,D). What, then, might be a plausible population-level explanation for the observed scaling of synaptic distributions?

**Figure 9:**
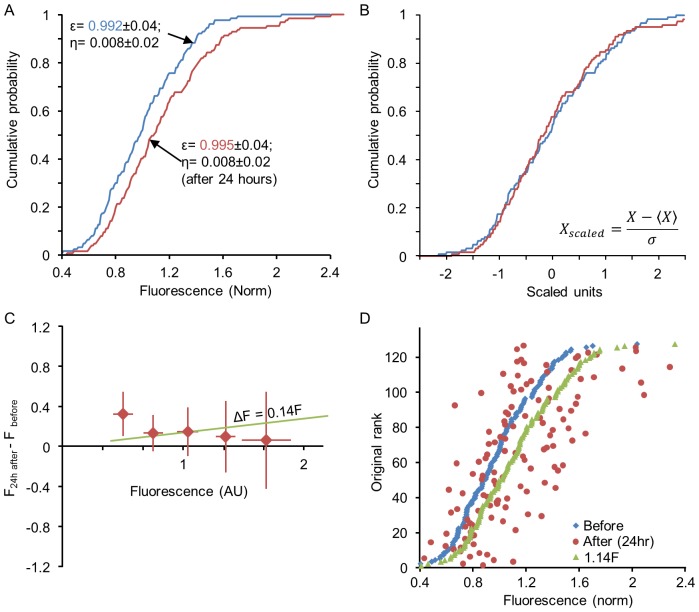
Distribution rescaling with individual rank-order shuffling in the Kesten process. The Kesten process provides a simple mechanism for population distribution rescaling without individual multiplication by a constant factor. Simulations were performed for 127 synapses (initial values taken from the synapses of Fig. 7). The synapses were first evolved for 24 hours (48 time points) with a Kesten process that preserved the original distribution. At this point 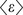 was slightly increased (from 0.992 to 0.995), and the trajectories were evolved for another 24 hours with the new parameters. (**A**) Distributions before (blue) and after (red) changing 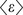. (**B**) Same distributions shown in (A) after scaling. (**C**) Changes in the fluorescence of individual synapses (*ΔF*) during the first 24 hours after changing 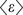 (averages and standard deviations of binned data). The green line represents the expected relationships between *ΔF* and *F* had sizes of individual synapses scaled through multiplication by 1.14 (the ratio of mean synaptic size before and after changing 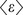. (**D**) Scaling without preserving rank order. Synapses were sorted according to their size before changing 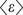 and plotted according to their original sizes (blue dots). The ‘sizes’ of the same synapses 24 hours after changing 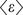 are shown as red dots. As in the experiments of Figs. 7 and 8, rank order is not preserved. The expected synaptic ‘sizes’, had scaling occurred multiplicatively, are shown as green dots.

Within the Kesten model, scaling emerges naturally from a change in the parameters of the underlying stochastic processes. Specifically, changes in *ε*-distributions and/or *η*-distributions can lead to a rescaling of the limiting distribution of synaptic sizes as shown in Fig. 6. Previous work has shown that, in plots such as that shown in Fig. 2, application of TTX affects strongly the slope whereas application of CCh noticeably alters the intercepts [4], [20], reflecting changes in the average values of the random variables *ε* and *η* respectively during these periods. Fig. 9 shows the same analysis as performed in Figs. 7 and 8 starting with the synapses of Fig. 7. These synapses were first evolved for 24 hours according to a Kesten process, using fixed statistical parameters. At the time of a simulated perturbation, one parameter of this process was altered; in this particular example, only 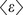 was changed, but similar results could be obtained by altering the *η*-distribution as well. A population-level rescaling results (Fig. 9A–B), but individual synapse size does not scale multiplicatively (Fig. 9C) and consequently, rank order is not preserved (Fig. 9D).

We thus conclude that changes in population synaptic distributions induced by two very different pharmacological manipulations, both of which induce scaling at the distribution level but not at the individual synapse level, are well captured by assuming that these manipulations modify the stochastic parameters underlying the Kesten process.

### Modeling the formation of new synapses as a Kesten process

**Figure 10:**
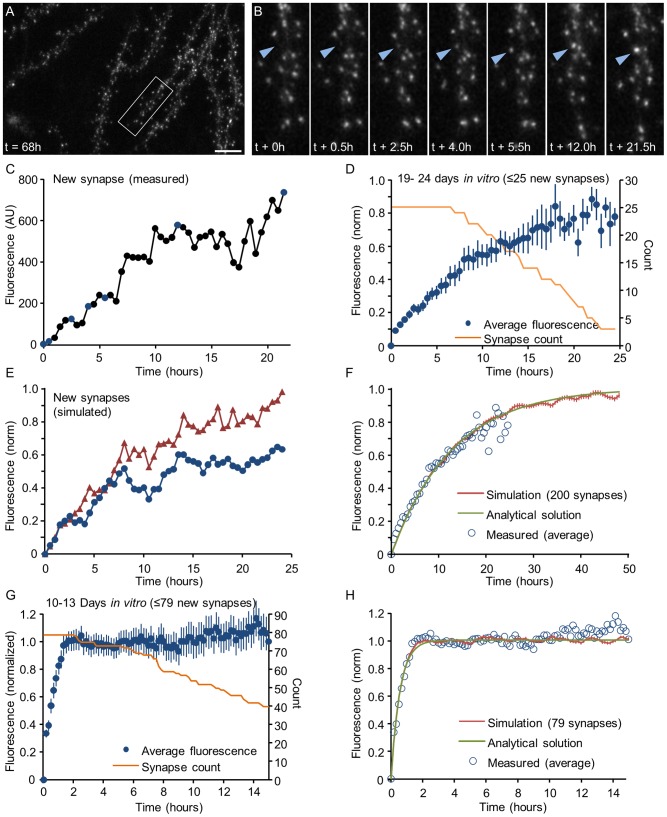
Kinetics of formation of new postsynaptic densities. **A,B**) The formation of a new PSD. Left panel: low magnification image of a dendrite 68 hours after the beginning of a time lapse session (started at 21 days *in vitro*). Right panels: gradual accumulation of PSD-95:EGFP at a new site (blue arrowhead). Bar: 10 µm. **C**) Time course of PSD-95:EGFP accumulation at the new site shown in **A**. The blue dots indicate the time-points of the images shown in B. **D**) Mean time course of new PSD formation in mature (>21 days in vitro) networks (average ± SEM). Data, pooled from 4 neurons, was aligned to the first time point at which a new PSD was observed. The fluorescence of each new synapse was normalized by subtracting the fluorescence value measured at its future location before a PSD was first detectable, and then divided by the background corrected mean fluorescence of the preexisting PSDs of that neuron. The number of new PSDs used to calculate the data points is shown as an orange line. **E**) Two simulated trajectories of new synapses, seeded with an initial value of 0.05 and evolved as a Kesten process with parameters 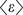  = 0.962±0.06 and 〈*η*〉 = 0.038±0.03 (Gaussian distributions). The resulting trajectories were normalized as the experimental data shown in D. **F**) Mean time course of synapse formation calculated analytically by equation (5) (green) and averaged over 200 simulated Kesten trajectories (red, average ± SEM) evolved and normalized as described in E. Open circles represent the experimentally measured data shown in D. **G**) Synapse formation in developing networks: mean time course of new PSD formation in developing networks (10–13 days *in vitro*; average ± SEM). Data, pooled from 3 neurons, was normalized as in D. The number of PSDs used to calculate the data points is shown as an orange line. **H**) Mean time course of synapse formation in developing networks calculated analytically based on equation (5) (green) and averaged over 79 simulated Kesten trajectories (red, average ± SEM). The parameters used for these simulations and calculations were 〈*ε*〉 = 0.74±0.06 and 〈*η*〉 = 0.26±0.03 (Gaussian distributions; 〈*η*〉 was constrained by 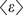 as explained in main text). Note that these reflect values for 10 minute steps (equivalent to 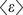  = 0.405 for half hour steps). Open circles represent the experimentally measured data shown in G.

Up to this point, the analysis focused on synapses that existed throughout the entire experiment (or analysis period). Cortical networks however, both *in vivo* and *in vitro*, also exhibit some degree of synaptic turnover, that is, the formation of new synapses and the elimination of others. The formation of a new excitatory synapse involves the formation of a new PSD, which can be detected as the accumulation of PSD-95:EGFP at a location at which no such accumulation was present before. An example of such an event is shown in Fig. 10A,B. Prior studies have suggested that this accumulation occurs in a gradual manner, but not necessarily monotonically, with periods of growth interspersed with pauses and even temporary periods of shrinkage [42], [43]. Fig. 10C shows how fluorescence accumulates with time at a site that was identified as a newly forming synapse. Can the dynamics of new PSD formation also be captured by a Kesten process?

To examine this possibility, we scrutinized time-lapse image series such as those shown in Fig. 1A–C for synapse formation events, and measured the evolution of PSD-95:EGFP fluorescence at these new synapses. Data were collected from spontaneously active networks (no pharmacological manipulations) after 3–4 days of baseline imaging. As seen in Fig. 10C, the increase of PSD-95:EGFP fluorescence was gradual, not entirely monotonic and quite protracted. To pool data from multiple occurrences of synapse formation, data was first temporally aligned to the first time point at which a new synapse was detectable. Note that as new synapses appeared at different times during time-lapse sessions of finite duration, this alignment resulted in synaptic trajectories of varying lengths. When data for all new synapses (n = 25, 4 neurons from 3 experiments) was normalized and pooled (see legend of Fig. 10), the average time course of PSD formation was obtained (Fig. 10D). We then generated simulated trajectories for 200 synapses based on a Kesten process, using an *ε*-distribution (and an *η* distribution constrained by 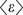 such that 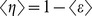 as explained above) that best fit the experimental data. Two exemplary trajectories shown in Fig. 10E appear qualitatively similar to typical trajectories measured in experiments, such as the one shown in Fig. 10C.

Plotting the average time course for the simulated data revealed that the experimental data could be described very well by a Kesten process (Fig. 10F). Interestingly, the values of 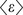 which provided the best fits were slightly smaller (0.962) than estimates obtained for established synapses in the same neurons. The observed difference indicates that the molecular dynamics associated with new synapse formation are somewhat faster than those occurring at established synapses, in line with a recent comparison of PSD-95:EGFP fluorescence fluctuations at stable and transient dendritic spines *in vivo* [24].

Because the initial size of a new synapse is close to zero, the average trajectory of a growing synapse can be approximated as a sum of a geometric series:
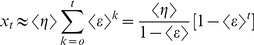
(6)


Under our normalization this is simply 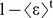, thus giving an exponential function with a typical timescale 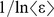
≅13 h. As shown in Fig. 10F, calculating equation (6) for the same values of 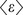 (and 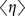) used in the simulations resulted in an excellent fit with the experimental and simulated data.

The average growth trajectory shows that the time course of PSD-95:EGFP accumulation at new sites occurred over many hours. This is much slower than the time course (1–2 hours) previously reported for PSD-95:EGFP accumulation in cultured hippocampal neurons at 8–12 days *in vitro* [42] but in good agreement with the time course of synaptic maturation measured in the barrel cortex of adult (>1 month old) mice [44], [45]. This difference may relate to the different developmental stage of the networks used in these studies. To examine this possibility, we measured PSD-95:EGFP accumulation at new sites in cortical networks grown and imaged in an identical fashion to those described throughout this study, except that here, week-long imaging sessions were initiated at days 9–10 *in vitro* instead of days 18–21 (this dataset is mentioned briefly in [4], and is exemplified in Video S1 in that study). We found that PSD-95:EGFP accumulation at new sites at days 10–13 *in vitro* (79 synapses, 3 neurons) was dramatically faster (Fig. 10G), concurring with rapid spine maturation in cultured slices of similar age [46]. Here too, the data could be very well fit to simulated trajectories based on a Kesten process as well as to the analytical approximation of equation (6) (Fig. 10H), except that in this case, the values of 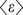 required for such fits were radically smaller (∼0.405) than those used so far (Figs. 4,5,9). This indicates that synaptic molecular dynamics during early developmental stages are faster than those occurring later on, in agreement with the extraordinary axonal and dendritic dynamism and high synapse formation and elimination rates observed in such networks (compare Video S1 in [4], to Video S1 in [20]; for review, see [23], [47]).

## Discussion

We have proposed a population dynamics approach for studying synapse remodeling dynamics based on a stochastic model known as the Kesten process. The basic premise of our approach is that synaptic size evolves over time due to a combination of multiplicative and additive processes, in which both multiplicative factors (*ε_t_*) and additive components (*η_t_*) are effectively stochastic and taken from distributions that are parametrically affected by physiological signals. We show that this seemingly simple model can generate rich dynamics which are qualitatively similar to the remodeling dynamics of preexisting and new synapses measured in long-term time-lapse experiments. Specifically, both the shape and the long-term stability of experimentally-measured synaptic size distributions are captured remarkably well by the Kesten process, and these properties are relativity insensitive to the details of the particular underlying *ε*- and *η*- distributions. External perturbations applied to the network affect mainly the *scale* of synaptic distributions while their shape remains intact. At the same time, the sizes of individual synapses exhibit stochastic changes with no deterministic transformation caused by the perturbation. These characteristics of synaptic distribution rescaling are congruent with a Kesten process in which *ε*- and *η-* distributions are altered by the external signals, providing a picture in which synaptic scaling at the population level coexists with disordered fluctuations at the individual synapse level.

### Robustness of the Kesten process

In the approach taken here the dynamics of the single synapse were assumed to reflect the integrated result of many microscopic processes, with their unknown dynamics represented by effectively random variables. As we have no prior knowledge of the statistical properties of these effective variables, an important consideration is the model's sensitivity to these statistics. We have found that the Kesten process shows a high degree of robustness with respect to these statistics: the shape of the distribution is completely insensitive to the properties of the additive variable (Fig. 6); the insensitivity of the distribution *tail* is ensured by the Kesten theorem, and our results extend this to show insensitivity of the distribution shape in the entire range. The dependence on the multiplicative variable is weak, and here too, the known dependence of the tail on its properties seems to apply to the entire distribution shape. These properties render the Kesten model an attractive candidate for effective modeling of synapse size dynamics.

Why is the Kesten process so generic and robust in its properties? Let us consider some global properties of the neural network in which the synapses are embedded. We know that the system is homeostatic, adaptive, and maintains itself around a stable state for some length of time while still fluctuating around it. Therefore the effective single-synapse dynamics must contain a “restoring” component in addition to an additive random component. If the value of the restoring component is allowed to be random as well, then to first (linear) approximation the Kesten process is obtained (see Text S1 for details). This proximity to a stable self-organized state may be at the core of the robustness of this model.

An interesting result supporting the emergence of Kesten-like dynamics as a result of network self-organization can be found in recent simulations which included the realization of multiple plasticity mechanisms [48]. In these simulations the effective dynamics of individual synapse were computed directly, and it was found that a multiplicative element in these dynamics emerged although it was not explicitly incorporated into the ingredients of the simulation. Thus, notwithstanding the debate concerning the additive or multiplicative nature of synaptic changes, their embedding in a network with both positive and negative feedback resulted in effectively stochastic changes that were both additive and multiplicative. It is worth noting in this regard that the Kesten process is, arguably, the simplest stochastic model that includes a state-dependent component (*ε_t_x_t_*) and a state-independent component (*η_t_*); other models, in which the dependence on *x_t_* is nonlinear, may be equally plausible.

### A statistical model for synapse dynamics and its relation to microscopic processes

Why should synapse dynamics be described by a statistical model, when so much is known about synaptic plasticity? A useful analogue in this respect is the description of neurotransmitter release as a stochastic process, modeled according to well-known statistical models, namely binomial or Poisson processes [49], [50]. Here synaptic vesicle release is characterized by a small number of parameters such as the number of release sites and the probability of release, both of which can vary as a function of history and stimuli in the network. Thus statistical models provide a compact, useful description which allows their parameters to change in response to physiological signals and perturbations. In contrast to this accepted statistical view of neurotransmitter release, *changes* in synaptic strength are usually described by deterministic rules that depend on detailed firing patterns of the connected neurons. The existence of a simple statistical model that reliably captures many aspects of the dynamics exhibited by individual synapses and synaptic populations is thus an interesting finding as it extends the stochastic view of the synapse to the realm of synaptic plasticity and tenacity.

The formulation of synaptic dynamics as a compact, low dimensional statistical model, essentially a combination of multiplicative and additive components, would seem to invite attempts to map each component to a specific biophysical process. For example, *ε_t_* might be considered to represent a rate constant in a first order reaction in which synaptic molecule loss (and accumulation) rates are proportional to synapse size [51]. Similarly, *η_t_* might be viewed as an additive process related to diffusion (or synthesis) of scarce synaptic molecules. Such specific mappings imply that synaptic remodeling is ultimately dictated by a very small number of dominant processes, with the rest of the molecules and processes playing only secondary or modulatory roles. Indeed, it has recently been suggested that in spite of the hundreds of molecules and processes implicated in Long Term Potentiation, this form of synaptic plasticity mainly depends on a very small number of factors, such as an adequate pool of surface glutamate receptors [52].

Alternatively, the insensitivity of the model to many underlying details may suggest that it is better viewed as an effective description of a large collection of processes, generally correlated with one another, combining in such a manner that their overall outcome is effectively a sum of multiplicative and additive variables. If this is the case, the relative insensitivity to underlying details (as exemplified in Fig. 6), indicates that it may not be possible, even in principle, to “reverse engineer” the population dynamics in order to infer their underlying microscopic processes. Interestingly, a similar biological buffering effect was suggested to underlie protein distributions measured in cells and to induce universal distributions across microorganisms and conditions [36]. We propose that the general question of the relation between multiple correlated microscopic stochastic processes and emergent behavior at the population level of organization poses fundamental questions in Neuroscience as well as in cell biology that merit further investigation, both experimental and theoretical.

### Previous models

Several groups have recently addressed the modeling of synaptic size dynamics by stochastic processes; we mention here two notably different approaches and compare them to ours. In the work of Yasumatsu and coworkers (2008) spontaneous synaptic size fluctuations were modeled by a generalized Fokker-Planck equation. Under this framework, different assumptions on the dependence of the moments on the synaptic volume lead to different distributions; thus to fit the data in various conditions (for example inhibitors), separate realizations of the model are needed. This reflects the fact that in the Fokker-Planck equation essentially any distribution can be obtained by assuming the appropriate potential and a Gaussian noise term. However there is no justification to choose a particular potential; moreover the steady-state distribution is highly sensitive to this choice.

A second approach was proposed by Loewenstein and coworkers (2011) in which spine remodeling is viewed as a purely multiplicative process, such that the log of the spine size is a sum of two Ornstein–Uhlenbeck processes and a white noise component. This model provides a good fit to the spine-size distribution and the timescales of the two processes can be fit from the correlation function, but is hard to justify biophysically beyond its successful fitting results. It should be noted that with finite data sets, broad distributions can often be fit equally well to several different functions. The reason is that the tails, which distinguish between different skewed distributions, are poorly sampled. Indeed our data can also be described by a log-normal distribution, as illustrated in Figure S3. Interestingly, the low end of the distribution seems to be better described by the Kesten model than by the log-normal distribution (Fig. S3 C,D).

The non-uniqueness of steady-state distributions in determining underlying stochastic models has been raised also in other areas of biophysics [36], [53]. It highlights the need for experiments that introduce perturbations and measure the system's transient dynamics, in addition to steady state measurements. The merit of different models should then be assessed based on properties other than fits to distributions. In this regard the Kesten model framework has two advantages: First, the inherent rescaling symmetry of the Kesten distribution under a change of underlying microscopic random variables, reflects nicely the measured property of synaptic distribution rescaling in response to perturbations; second, a compound process with two types of accumulation – sum and product – can be justified as a generic, effective stochastic description for a large number of correlated processes.

### Functional implications of stochastic synaptic dynamics

Operationally, if one accepts the premise that synaptic remodeling is governed by a vast number of complex, interconnected and to some extent, intractable molecular processes, then the model proposed here may provide a useful framework for characterizing synaptic dynamics and predicting their outcome irrespective of underlying details. However, it is also worth considering the functional implications of this perspective.

In the context of learning theories, synapse remodeling has been traditionally viewed as a process dictated by physiological signals, and interpreted in the context of synapse-specific “learning rules” or global homeostatic processes. Such learning rules are expected to ultimately provide a link between individual synapse behavior and the systems property, namely learning and memory. The approach proposed here, to view synaptic dynamics as stochastic, seems to represent a major departure from this deterministic view. Several caveats regarding these two views should be considered, however.

Starting with the data used here, it is important to note that these were obtained in networks devoid of external input, and thus, perhaps, in the absence of strong instructive forces. Moreover, the forms of spontaneous activity observed in these networks are strongly reminiscent of cortical activity forms observed during deep sleep and anesthesia (e.g. [20], [54]). Thus, it may be argued that the remodeling dynamics observed and analyzed here might be more representative of “baseline” synaptic dynamics in the absence of meaningful input. However, it is noteworthy that fluctuations in PSD size [24], [55], spine volume [3], [56] and presynaptic bouton size [10] of comparable magnitude are also observed *in vivo*. For example, recent measurements of synaptic size fluctuations in cortical neurons of 8 week old mice based on PSD-95:EGFP fluorescence (as done here) reveal that the magnitude of such fluctuations is very considerable (∼48% change on average over periods of 0.25 to 4 days [24]; for comparison, changes induced in organotypic rat hippocampal slice cultures by protocols that drive long-term potentiation are ∼33% on average [25]). While these spontaneous fluctuations might be driven by the animal's behavioral experiences, it should be noted that in cell culture [2], [4] and in organotypic cultures [5], fluctuations persist even when all activity is blocked. It would thus seem that our empirical observations are not limited to the setting of cell culture; furthermore, if spontaneous size fluctuations are as large as the aforementioned studies suggest, a framework which brings into account baseline size dynamics is needed.

Second, suggestions for the governance of synaptic remodeling by *selective*, rather than instructive processes have been previously put forward (for example [4], [23], [56]–[60]). The underlying notion is that remodeling occurs stochastically, and that favorable changes are selected by physiological signals. According to this view, even though synaptic remodeling is driven by stochastic processes, on the whole it is also governed by instructive processes in the form of feedback and reinforcement.

Third, it is assumed that physiologically-relevant manipulations might lead to parametric changes in *ε*- and *η*- distributions; such changes could occur at select sets of synapses (for example, synapses that undergo directed potentiation or depression) or at larger sets of synapses (in response to global changes in input or activity level, for example). Thus, while individual synapse remodeling may appear to be effectively stochastic, statistical properties of select or large synaptic populations may still change in a manner determined by signals in the environment [32]. It remains to be seen how sizes of such populations relate to the relatively small number of connections formed between any two neurons [61] and at what organizational level, if any, invariance and determinacy emerge [62].

## Materials and Methods

### Time-lapse imaging of excitatory synapses in primary cultures of cortical neurons

The data presented here is mainly taken from two prior studies ([4], [20]) and [63]. Detailed descriptions of the methodologies used during those studies, which can be found in the aforementioned references, are summarized briefly below.

Primary cultures of rat cortical neurons were prepared from cortices of 1–2 days-old rats (either sex) which were dissected, dissociated and plated on thin glass Multielectrode array (MEA) dishes (MultiChannelSystems MCS, Reutlingen, Germany). Cells were plated in media containing minimal essential medium (MEM, Sigma), 25 mg/l Insulin (Sigma), 20 mM Glucose (Sigma), 2 mM L-Glutamine (Sigma), 5 µg/mL Gentamycin sulfate (Sigma) and 10% NuSerum (Becton Dickinson Labware, Bedford, Massachusetts, United States). Preparations were then transferred to a humidified tissue culture incubator and maintained at 37°C in a gas mixture of 5% CO_2_, 95% air. Half the volume of the culture medium was replaced 3 times a week with feeding media, essentially identical to seeding media, except for the omission of NuSerum, lower L-Glutamine concentrations (0.5 mM) and the addition of 2% B-27 supplement (Invitrogen, San Diego, CA).

Expression of enhanced green fluorescent protein (EGFP)-tagged PSD-95 was carried out by transduction on day 5 in-vitro with third generation lentiviral particles prepared and used as described elsewhere [20].

Imaging was performed on a custom designed confocal laser scanning microscope using a 40×, 1.3 N.A. Fluar objective (Zeiss). The system was controlled by software written by one of us (NEZ) and includes provisions for automated, multisite time-lapse microscopy. The MEA dishes were mounted on a commercial 60-channel headstage/amplifier (MultiChannelSystems) attached to the microscope's motorized stage, and covered with a custom designed cap containing inlet and outlet ports for perfusion media and air mixtures, a reference ground electrode and a removable transparent glass window. The MEA dish was continuously perfused with feeding media (described above) at a rate of 2.5–5 ml/day by means of a custom built perfusion system based on an ultra-slow peristaltic pump (Instech Laboratories Inc., Plymouth Meeting, PA, USA) using an imbalanced set of silicone tubes. The tubes were connected to the dish through appropriate ports in the cap. A 95% air/5% CO_2_ mixture was continuously streamed into the dish at very low rates through a third port with flow rates regulated by a high precision flow meter (Gilmont Instruments, IL, USA). The base of the headstage/amplifier and the objective were heated to 37°C and 36°C respectively using resistive elements, separate temperature sensors and controllers, resulting in temperatures of 36–37°C in the culture media.

EGFP was excited using the 488 nm line of an argon laser. Fluorescence emissions were read through a 500–545 nm bandpass filter (Chroma Technology, Brattleboro, VT). Time-lapse recordings were usually performed by averaging six frames collected at each of 7 to 26 focal planes spaced 0.8–1 µm apart. All data were collected at a resolution of 640×480 pixels, at 12 bits/pixel, with the confocal aperture fully open. Data was collected sequentially from up to 12 predefined sites, using the confocal microscope robotic XYZ stage to cycle automatically through these sites at intervals of 10 minutes (Fig. 10G,H), 60 minutes (Fig. 2D) 25 minutes (Fig. 2B,C) or 30 minutes (all other data). Focal drift during the experiment was corrected automatically by using the microscopes' “autofocus” feature.

Experiments performed in chemically fixed neurons [4], were performed as described above except that here preparations were first fixed with 4% paraformaldehyde in phosphate buffered solution (PBS), washed several times with PBS, placed in growth medium, mounted on the microscope, heated, connected to the sterile air and perfusion systems and imaged at 30 minute intervals as described above for live neurons.

### Chemical perturbations

Tetrodotoxin (TTX, Alomone labs, Israel) and CCh (Carbachol, Carbamoylcholine) were applied by diluting them into 100 µL of medium drawn from the culture dish while on the microscope. The mixture was subsequently returned to the dish and mixed gently. Applications to the dish were complemented by simultaneous addition to the perfusion media. Final concentrations in the dish and media were 1 µM (TTX) and 20–50 µM (CCh).

### Image analysis

Data analysis of image time series was performed using custom written software (“OpenView”) written by one of us (NEZ). Special features of this software allow for automated/manual tracking of objects in 3D time series of confocal images as described elsewhere [20]. 8×8 or 9×9 pixel (∼1.3×1.3 µm) areas were then centered on the centers of such objects and mean pixel intensities within these areas were obtained from maximal intensity projections of Z section stacks. For tracking identified puncta, areas were placed initially over all puncta and then a smaller subset (typically 100–150 per site) was thereafter tracked. For tracking newly forming puncta, new puncta were manually identified in time-lapse movies and then tracked from the movement of their appearance for as long as they were present, as long as tracking was unambiguous, or the end of the time series was reached. As the reliability of automatic tracking was not absolutely perfect, all tracking was verified and, whenever necessary, corrected manually. Puncta for which tracking was ambiguous were excluded.

Microscopy images for Figs. 1 and 10 were processed by contrast enhancement and low-pass filtering using Adobe Photoshop.

All data were exported to Matlab or Microsoft Excel and analyzed using custom written scripts. Final graphs were prepared using Excel. All final figures were prepared using Microsoft PowerPoint.

## Supporting Information

Dataset S1 This Microsoft Excel file contains the raw fluorescence values measured from individual synapses. Each page is marked by the corresponding figure number and rows and columns are titled with the relevant variable (e.g. “time”, “synapse #”, etc.).
(XLSX)

Figure S1Measurement noise analysis. (**A**) A single neuron expressing PSD-95:EGFP that was chemically fixed before the experiment (as explained in Materials and Methods) and imaged for 43 hours at 30 min intervals. The right hand side shows a higher magnification of the region enclosed in a rectangle at 5 time points. All images are maximal intensity projections of 9 images collected at 9 focal planes spaced 0.8 µm apart. Bars: Left - 10 µm; right 5 µm. (**B**) PSD-95:EGFP fluorescence levels of 10 arbitrary synapses from live neurons. Top – raw data; bottom – after filtering with a 5 time-point low pass filter. (**C**) PSD-95:EGFP fluorescence levels of 10 arbitrary synapses from fixed neurons. Top – raw data; bottom – after filtering with a 5 time-point low pass filter. (**D**) Means and standard deviations of Coefficient of Variations (CV) of PSD-95:EGFP fluorescence values measured for each synapse over a 24 hour period (live neurons: 1087 synapses; fixed neurons: 1067 synapses). Values are shown for CVs computed for raw fluorescence measurements and for the same synapses after filtering the fluorescence measurements with a 5 time-point low pass filter. (**E**) Estimating 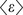 from all possible pairs of measurements made from each synapse from all synapses in the live neuron data set (1087) at different time-step intervals ranging from 1 step (0.5 hour) to 12 time steps (6 hours). Note that the estimate of 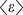 improves with longer time intervals, as the contamination by measurement noise become gradually less significant. This improvement is much more apparent for the unfiltered data but still observable even after filtering the data with a 5 time-point low pass filter.(TIF)

Figure S2Validation of the Kesten model. (**A**) Testing the estimation procedure on two halves of the data. The estimation for 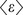 described in Fig. 4 was performed on half of the synapses and the resulting line shown here was based on the other half of the data. (**B,C**) In the Kesten process, variance of the residuals in a linear fit of a one-step scatter-plot (i.e. plotting *x_t_*_+1_ as a function of *x_t_* for all synapses at all time-points) should lie on a parabola whose second order coefficient reflects the variance 〈*δ* ^2^ *ε*〉 = 〈(*ε*-〈*ε*〉)^2^〉. The first order coefficient should be zero if ε and η are independent. The analysis shown here was performed on (**B**) experimental data (1087 synapses) and (**C**) simulated data (same number of points as data; same parameters as in Fig. 5). Although these one-step plots should be treated with caution because of measurement noise (Fig. S1), the fits for the data and simulations are generally similar.(TIF)

Figure S3 “Log-normality” of the data. Comparison of fits of a synaptic size histogram to a log-normal distribution and to a Kesten distribution. (**A**)**–**(**C**) Synaptic histograms (blue circles) and corresponding fits to Kesten simulation (red line) and log-normal distribution (green line), in three different axes systems. The semi-logarithmic axes highlight the distribution tail. The double logarithmic axes highlights the power-law-like behavior of the tail; the dashed line is a power law of (−5). Note that although the log-normal distribution behaves asymptotically as a power law of (−1), the region relevant to the data is still far from the asymptotic regime. (**D**) A scatter plot of the Cumulative Probability Density (CDF) avoids the need to bin the data and highlights the left hand tail of small synapses, indicating that the Kesten model provides a slightly better fit in this regime.
(TIF)

Text S1 Heuristic justification for the Kesten process. We develop an approximate argument based on expansion around as statistically stable steady state of a complex system, to suggest how the Kesten process might arise as effective dynamics projected onto a single degree of freedom in such a system.
(PDF)

Video S1 Evolution of slopes and intercepts in experimental data. Changes in the slope, intercept and goodness of fit (R-square) over a period of 24 hours (48 time steps) when the size of each synapse is compared to its size at time step 1. Note the gradual decrease in the slope, the gradual increase in the intercept value, and the gradual reduction in R-square values. Note the tendency of small synapses to grow larger, the tendency of large synapses to grow smaller, and the apparent rotation of the regression line around an imaginary pivot point at x = 1. Same data as in Fig. 4.
(AVI)
