# Supplementary figures and images for: Synaptic Size Dynamics as an Effectively Stochastic Process
Source: PLoS Comput Biol. 2014 Oct 2;10(10):e1003846. doi: 10.1371/journal.pcbi.1003846 (PMC4183425; doi:10.1371/journal.pcbi.1003846)

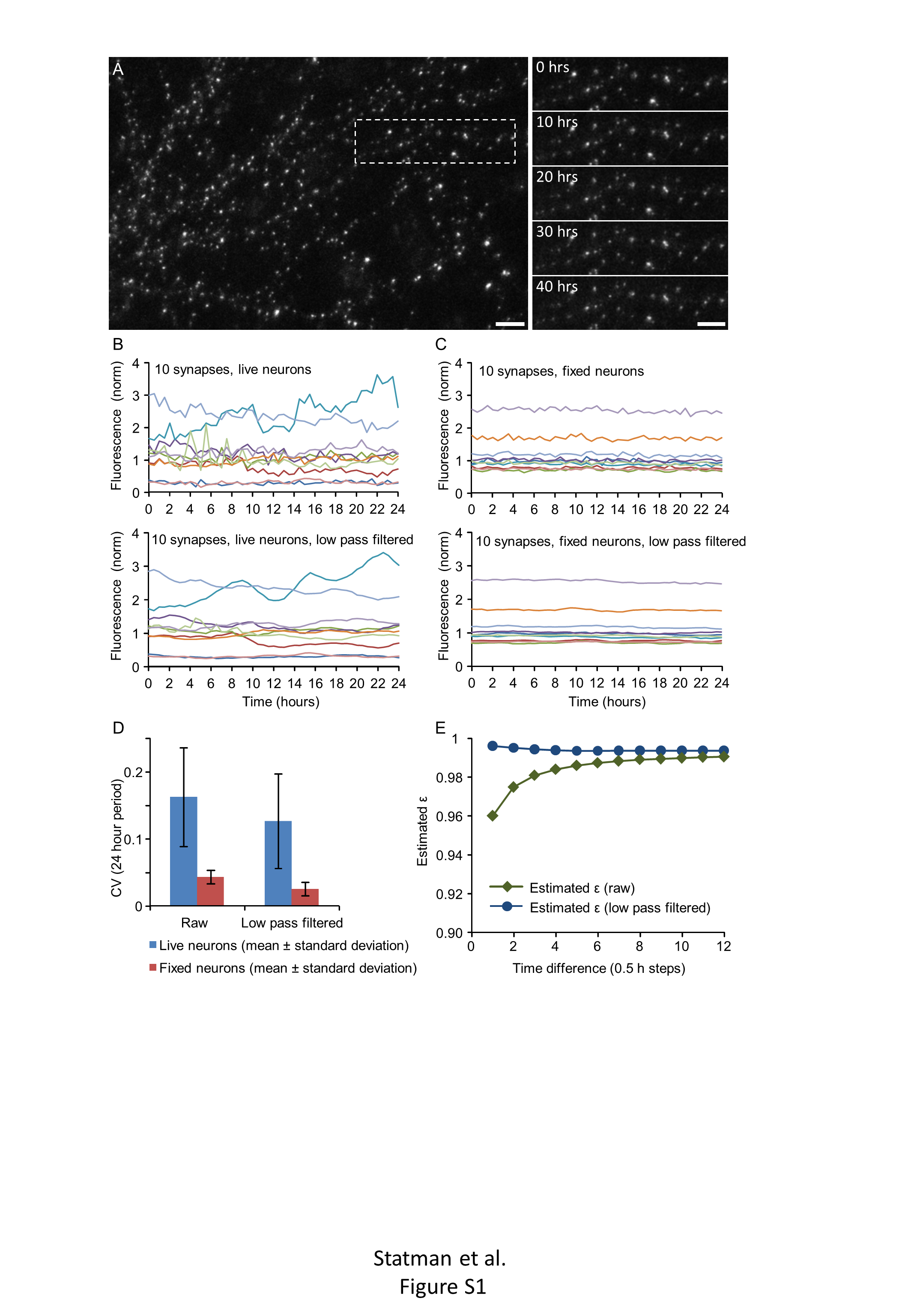

Supplement: Figure S1 — Measurement noise analysis. (A) A single neuron expressing PSD-95:EGFP that was chemically fixed before the experiment (as explained in Materials and Methods) and imaged for 43 hours at 30 min intervals. The right hand side shows a higher magnification of the region enclosed in a rectangle at 5 time points. All images are maximal intensity projections of 9 images collected at 9 focal planes spaced 0.8 µm apart. Bars: Left - 10 µm; right 5 µm. (B) PSD-95:EGFP fluorescence levels of 10 arbitrary synapses from live neurons. Top – raw data; bottom – after filtering with a 5 time-point low pass filter. (C) PSD-95:EGFP fluorescence levels of 10 arbitrary synapses from fixed neurons. Top – raw data; bottom – after filtering with a 5 time-point low pass filter. (D) Means and standard deviations of Coefficient of Variations (CV) of PSD-95:EGFP fluorescence values measured for each synapse over a 24 hour period (live neurons: 1087 synapses; fixed neurons: 1067 synapses). Values are shown for CVs computed for raw fluorescence measurements and for the same synapses after filtering the fluorescence measurements with a 5 time-point low pass filter. (E) Estimating from all possible pairs of measurements made from each synapse from all synapses in the live neuron data set (1087) at different time-step intervals ranging from 1 step (0.5 hour) to 12 time steps (6 hours). Note that the estimate of improves with longer time intervals, as the contamination by measurement noise become gradually less significant. This improvement is much more apparent for the unfiltered data but still observable even after filtering the data with a 5 time-point low pass filter. (TIF) [file pcbi.1003846.s002.tif]

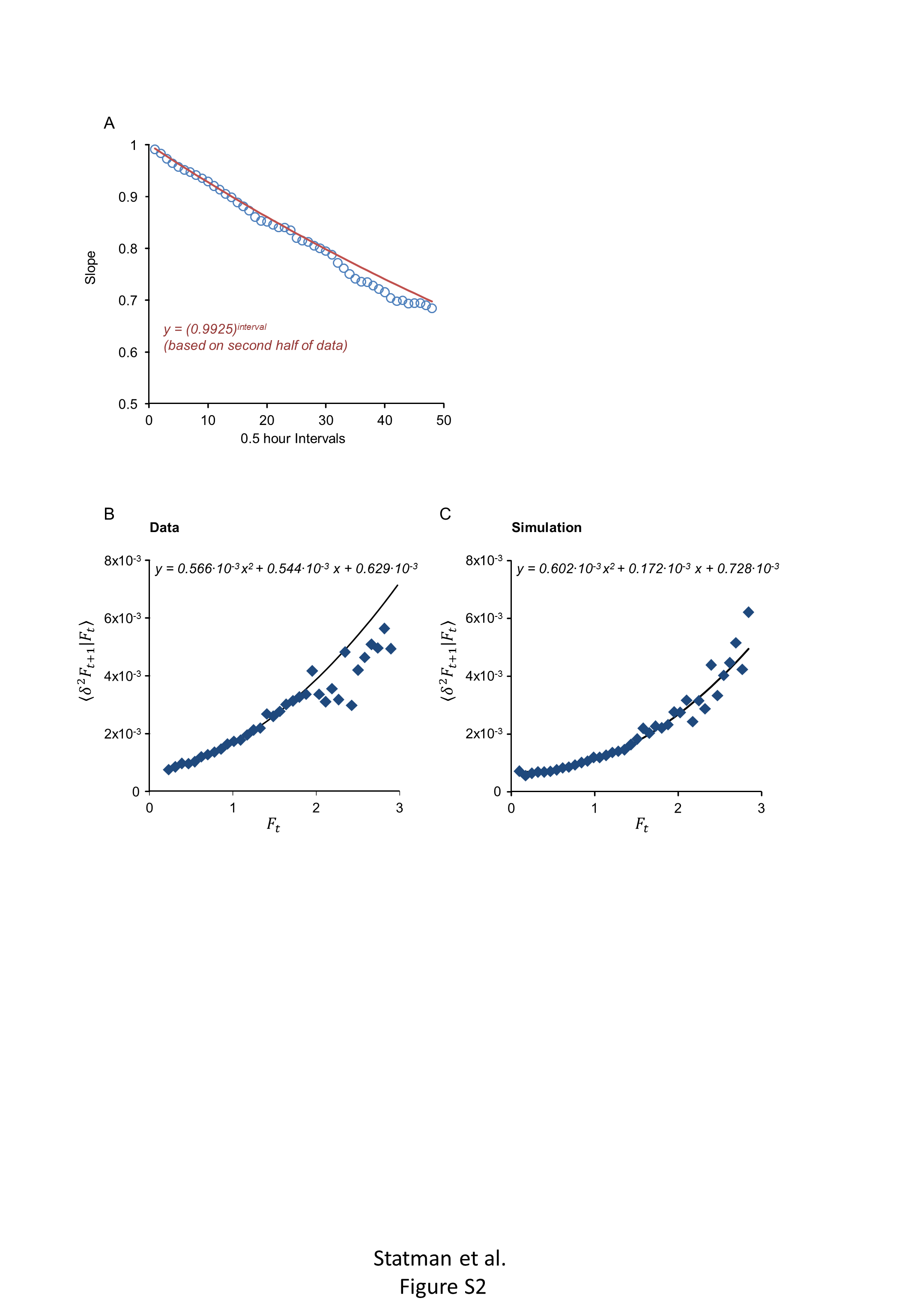

Supplement: Figure S2 — Validation of the Kesten model. (A) Testing the estimation procedure on two halves of the data. The estimation for described in Fig. 4 was performed on half of the synapses and the resulting line shown here was based on the other half of the data. (B,C) In the Kesten process, variance of the residuals in a linear fit of a one-step scatter-plot (i.e. plotting xt +1 as a function of xt for all synapses at all time-points) should lie on a parabola whose second order coefficient reflects the variance 〈δ 2 ε〉 = 〈(ε-〈ε〉)2〉. The first order coefficient should be zero if ε and η are independent. The analysis shown here was performed on (B) experimental data (1087 synapses) and (C) simulated data (same number of points as data; same parameters as in Fig. 5). Although these one-step plots should be treated with caution because of measurement noise (Fig. S1), the fits for the data and simulations are generally similar. (TIF) [file pcbi.1003846.s003.tif]

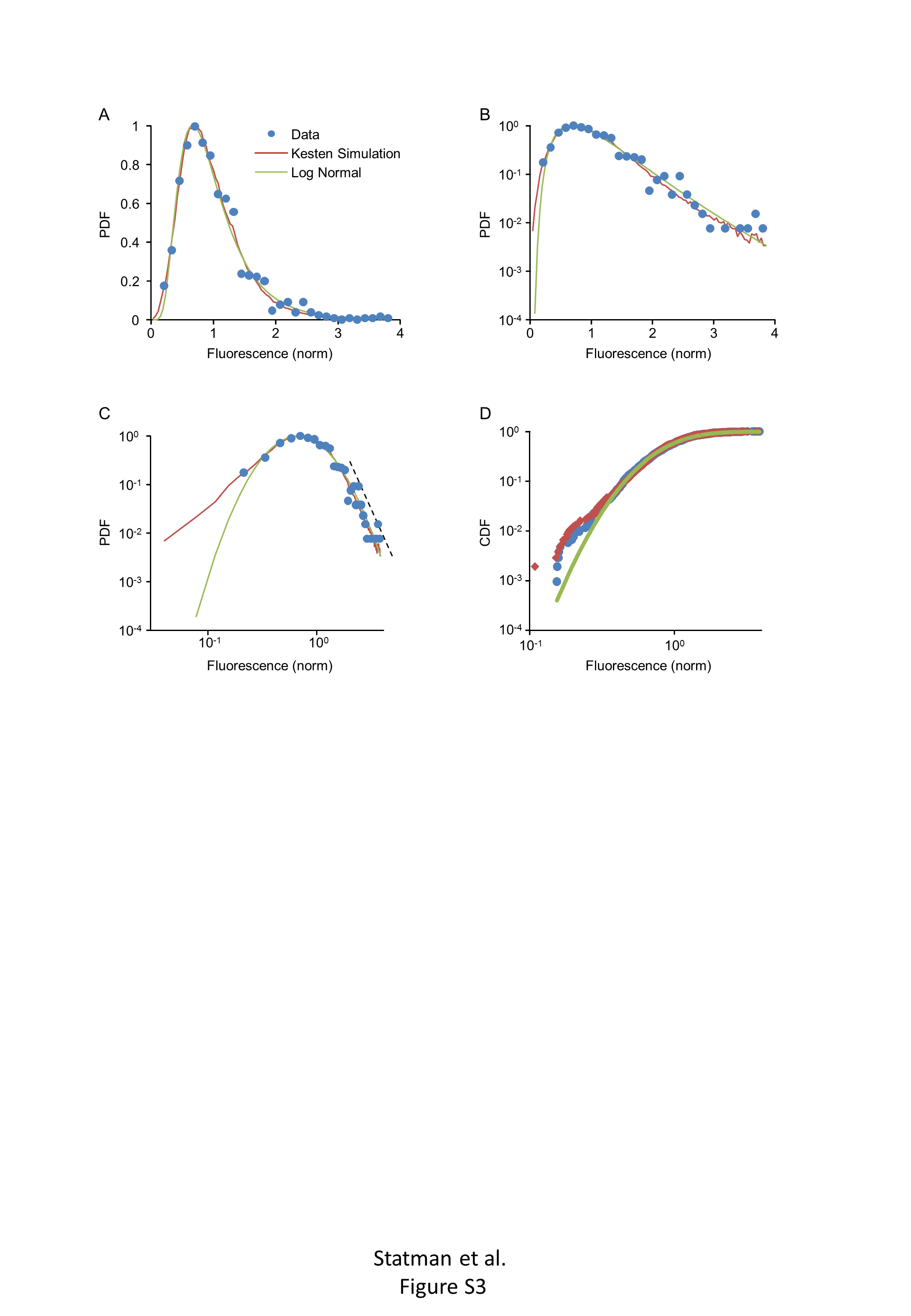

Supplement: Figure S3 — “Log-normality” of the data. Comparison of fits of a synaptic size histogram to a log-normal distribution and to a Kesten distribution. (A)–(C) Synaptic histograms (blue circles) and corresponding fits to Kesten simulation (red line) and log-normal distribution (green line), in three different axes systems. The semi-logarithmic axes highlight the distribution tail. The double logarithmic axes highlights the power-law-like behavior of the tail; the dashed line is a power law of (−5). Note that although the log-normal distribution behaves asymptotically as a power law of (−1), the region relevant to the data is still far from the asymptotic regime. (D) A scatter plot of the Cumulative Probability Density (CDF) avoids the need to bin the data and highlights the left hand tail of small synapses, indicating that the Kesten model provides a slightly better fit in this regime. (TIF) [file pcbi.1003846.s004.tif]
