# Supplementary material for: Synaptic Size Dynamics as an Effectively Stochastic Process
Source: PLoS Comput Biol. 2014 Oct 2;10(10):e1003846. doi: 10.1371/journal.pcbi.1003846 (PMC4183425; doi:10.1371/journal.pcbi.1003846)
Supplement: Text S1 — Heuristic justification for the Kesten process. We develop an approximate argument based on expansion around as statistically stable steady state of a complex system, to suggest how the Kesten process might arise as effective dynamics projected onto a single degree of freedom in such a system. (PDF) [file pcbi.1003846.s005.pdf]

Synaptic Size Dynamics as an Effectively Stochastic Process
Supporting information

Text S1: Effective Stochastic Dynamics

Much attention has been devoted to the question of the stability of synaptic structures in terms of dynamical models [1,2]. Here we aim to sketch a justification for our simple stochastic model by general dynamical considerations assuming the existence of an underlying mechanism that ensures stability. We assume that spatial degrees of freedom and interactions among different molecular species can be integrated over time, to describe the nonlinear dynamics of synaptic size as a general one-dimensional dynamical system:

$$\frac{dx}{dt} = f(x)$$

where x is the synaptic size and the function f, generally dependent on x, describes effectively all these interactions. This is a deterministic equation, and we shall introduce a stochastic component to the equation to account effectively for many degrees of freedom that have been integrated out.

Near a "fixed point" of these dynamics, namely around a stable average synaptic size x_0, the deterministic derivative $f(x)$ must cross the x-axis with a negative slope:

$$f(x_0) = 0, \quad f'(x_0) < 0$$

This can be viewed as the most fundamental form of instantaneous "negative feedback" which ensures stability against perturbations. Now we define a discrete-time mapping from this dynamical system, which uses the linear approximation in the vicinity of the fixed point:

$$x(t+\Delta t) = x(t)\left(1 + f'(x_0)\Delta t\right) - f'(x_0)x_0\Delta t$$

Treating this dynamical equation as an average of the true dynamics, and introducing a random element in its parameters, one obtains for a given time interval a Kesten process. The average negativity of the derivative ensures that the random variables are in the regime of stability of the Kesten process (this is an approximate argument for small variance). The introduction of stochasticity to the equation is not a controlled approximation, but instead a heuristic argument reflecting the fact that this process is coupled to a large number of other processes whose details we cannot account for. The existence of an underlying fixed point

ensures that on average the multiplicative term is smaller than one, whereas the additive term is positive.

We note that in the classic treatment of Statistical Physics, a system with random fluctuations and a restoring force is described by the Langevin equation

$$\frac{dx}{dt} = -\gamma x + \eta(t)$$

Where $-\gamma x$ is a deterministic restoring force and η a random variable [3]. This description relies on a separation of timescales between the random fluctuations and the force acting on the particle and is justifiable as a description of a particle undergoing Brownian motion. The time-series resulting from this dynamic equation is also known as the Ornstein-Uhlenbeck process, usually defined with a Gaussian distribution for η, and the resulting steady-state probability distribution is correspondingly Gaussian. This property can be generalized, but then the steady-state distribution reflects the distribution of η. Previous work has used this model to describe synaptic size dynamics [4]; a logarithmic transformation was used to obtain the measured skewed synaptic distribution, and two such processes were added to account for two timescales in the measured correlation function. While this procedure adequately describes the data it is hard to justify the arbitrary logarithmic transformation on biophysical grounds.

In a more general setting if there is no basis for a timescale separation, it is hard to justify the construction of an equation which is partly determinstic and partly stochastic. In this case, both terms should be considered as fluctuating, a "multiplicative noise" arises in the equation and we arrive naturally at the Kesten process.

Some intuition as to the emergence of a multiplicative element in the dynamics from actual feedback mechanisms can be found from simulations on network with mutliple mechanisms of synaptic plasticity [5]. It was found that the effective dynamics of individual synapses were stochastic-like in nature and displayed a "rich gets richer" dynamics although the STDP rule implemented was additive. Moreover, a direct computation of the effective change in individual synapses as a function of current value showed a behavior typical of the Kesten process (J. Triesch, private communication). This can be explained by a combination of self-reinforcing and competitive mechanisms that together caused the network self-organization at a statistically stable state. This result provides an explicit example of the abstract steady-state and fluctuations around it described in this Appendix.

References

1. Haselwandter CA, Calamai M, Kardar M, Triller A, da Silveira RA (2011) Formation and Stability of Synaptic Receptor Domains. Physical Review Letters 106.
2. McAllister AM (2007) Dynamic aspects of CNS synapse formation. Annual Review of Neuroscience 30: 425-450.
3. van-Kampen NG (1992) Stochastic processes in physics and chemistry: North Holland.
4. Loewenstein Y, Kuras A, Rumpel S (2011) Multiplicative Dynamics Underlie the Emergence of the Log-Normal Distribution of Spine Sizes in the Neocortex In Vivo. J Neurosc 31: 9481–9488.
5. Zheng PS, Dimitrakakis C, Triesch J (2013) Network Self-Organization Explains the Statistics and Dynamics of Synaptic Connection Strengths in Cortex. Plos Computational Biology 9.

Legend to Dataset S1

This xls file contains the raw fluorescence values measured from individual synapses. Each page is marked by the corresponding figure number and rows and columns are titled with the relevant variable (e.g. "time", "synapse #", etc.).
